# Effect of the Reduction in Training Volume during the COVID-19 Era on Performance in 100-m and 400-m Freestyle Events in Greek Swimming Championships

**DOI:** 10.3390/sports10030040

**Published:** 2022-03-07

**Authors:** George Tsalis, Vassilis Mougios

**Affiliations:** 1 School of Physical Education and Sport Science at Serres, Aristotle University of Thessaloniki, 62110 Serres, Greece; 2 Laboratory of Evaluation of Human Biological Performance, School of Physical Education and Sport Science at Thessaloniki, Aristotle University of Thessaloniki, 54124 Thessaloniki, Greece; mougios@auth.gr

**Keywords:** COVID-19, detraining, swimming performance

## Abstract

The COVID-19 pandemic had a profound impact on almost all social activities, including sport participation. Swimming training was greatly reduced during the 2019–2020 and 2020–2021 seasons (by four and two months, respectively), which caused athletes and coaches to worry about performance in championships. The present study investigated the impact of COVID-19 lockdowns on the results of Greek swimming championships in the categories of 13 to 18 years of age during 2020 and 2021. Forty-one coaches were interviewed about the training process (satisfaction, duration of the training season, and daily training swimming distance), and the performance of swimmers in national championships over seven seasons (2014–2021) was analyzed. The duration of the training season and the daily swimming distance, as reported by the coaches, were lower during the 2019–2020 and 2020–2021 seasons, compared to the previous five seasons (*p* < 0.001). The number of swimmers who achieved qualifying times for the national championships during the COVID-19 era were similar to those in previous years. Comparisons of the times in the 100-m and 400-m freestyle events, in both genders, from 2015 through 2021, showed no significant differences except for the 400-m event in males, in which a lower performance was detected in 2021 compared to 2015 (by 2.7%, *p* = 0.001). In conclusion, two or four months of detraining during the COVID-19 era had a negative impact on the coaches’ evaluation of the training process, but there was no effect on the number of young swimmers who qualified for the national championships and a negligible effect on swimming performance in 100-m and 400-m freestyle events.

## 1. Introduction

The years 2020 and 2021 were the years of the “COVID-19 era”. Lockdowns on professional and social activities to protect public health took their toll on the training of competitive athletes in many countries, including swimmers in Greece. Because many aquatic facilities are indoor, the Hellenic Swimming Federation (HSF), in accordance with the Greek government’s safety protocols, allowed only a few swimmers, belonging to the national teams, to continue training. The remaining competitive swimmers, who regularly participated in national championships, stopped training. This resulted in lower training load and reduced training specificity, as also reported by Pla et al. [1].

Most coaches in Greece train swimmers that are 13 to 18 years old, according to HSF records. These coaches must design training programs that meet the needs of different ages, capabilities, and specificities. Coaches have more-or-less stable habits, or a “coaching style” [2], and usually place a strong emphasis on high-volume, low-intensity training [3,4]. This is because coaches and swimmers alike feel more comfortable, confident, and assured about upcoming swimming results when they perform high volumes, despite evidence that it is better to turn up the intensity and turn down the volume to improve performance [5].

During the 2019–2020 competitive season (the first of the COVID-19 era), swimmers in Greece stopped swimming training for two months, while, during the 2020–2021 season, swimming training stopped for four months. During the entire training seasons, most of the swimmers performed dry-land training, more so during the lockdowns (even through online guidance), to compensate for the lack of swimming training, as reported by the coaches.

As a result of these long detraining periods, swimmers and even more coaches became anxious about the reduction of what they considered the most important factor in training, i.e., volume, and conveyed these concerns to the authors on several occasions. Such concerns are justifiable as it is known that detraining periods of 4 to 10 weeks are detrimental to swimming performance [6,7]. Nonetheless, at the 2021 Greek swimming championships, many coaches expressed the feeling that their athletes had performed well relative to previous years. This spurred our interest in exploring whether such an impression was objectively justified. Thus, the aims of the present study were to investigate the impact of the COVID-19 restrictions on (i) the coaches’ evaluation of the training process and (ii) swimmers’ performance, as reflected in the results of the Greek national championships in the 100-m and 400-m freestyle events. As a reference, we considered the preceding five seasons (2014–2019) so as to minimize the effects of yearly fluctuations in the study parameters on our comparisons.

## 2. Materials and Methods

### 2.1. Coaches

To evaluate the training process over the seven seasons from 2014 to 2021, we asked 41 coaches (37 men and 4 women), aged 46 ± 10 years and with a training experience of 20 ± 10 years (mean ± SD) from Northern Greece, all of whom trained swimmers that participated in national championships, to complete a simple questionnaire describing the quality and quantity of training during each season, based on their records and recalls. The questionnaire included the following questions: (i) “How satisfied were you with the training process relative to the 2014–2015 season (on a scale of 0 to 100%)?”, (ii) “How many months did your swimmers train?”, (iii) “How many kilometers did your short-distance swimmers swim per day during training on average?”, and (iv) “How many kilometers did your middle-distance swimmers swim per day during training on average?”.

### 2.2. Swimmers

As is the case worldwide, most Greek competitive swimmers are between 13 and 18 years old. The following four categories have been established in the Greek national championships (in both sexes): 13 years, 14 years, 15 plus 16 years combined, and 17 plus 18 years combined. Data on swimmers’ performance in the 100-m and 400-m freestyle events in the Greek championships of these four categories for the years 2015 to 2021 were obtained from the HSF (https://koe.org.gr/koearchive/kolimvisi/apotelesmata, accessed on 1 September 2021). Only swimmers who had achieved the time standards set by the HSF participated in the 2015 to 2019 championships (the time standards remained the same per category and event during those years). However, because of the COVID-19 pandemic, the HSF did not apply this rule to the 2020 and 2021 championships; instead, they allowed all interested swimmers to participate. Nevertheless, we included only those who achieved the corresponding time standards in our analysis. For all swimmers, the records that were included in the analysis were the best of two rounds, namely the eliminatory and finals. Additionally, to facilitate social distancing, separate championships took place in Southern and Northern Greece in 2020 and 2021. The numbers of swimmers who were included in the analysis are presented in Table 1.

### 2.3. Statistical Analysis

The distribution of data was examined through the Shapiro–Wilk test. Because the distribution differed significantly from normal in most cases, analyses were performed through non-parametric tests, with the level of statistical significance set at α = 0.05. The coaches’ responses were compared across years through the Friedman test, with significant effects being followed up by the Wilcoxon test with Bonferroni correction. Swimmers’ records were compared across years through the Kruskal–Wallis H test, with significant effects being followed up by the Mann–Whitney U test with Bonferroni correction. Data were analyzed using SPSS 27.0 (SPSS, Chicago, IL, USA).

## 3. Results

### 3.1. Training and Competitive Seasons before and during the COVID-19 Era

**Figure 1:**
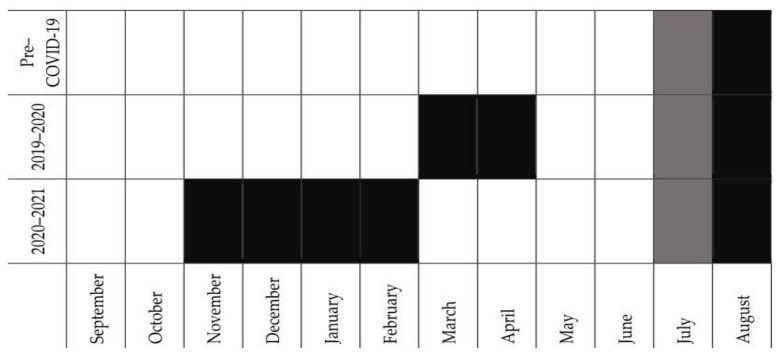
Training and competitive periods before and during the COVID-19 era. Open boxes indicate months of training, black boxes indicate months of detraining, and grey boxes indicate the months in which national swimming championships took place.

During the 2019–2020 competitive season (the first of the COVID-19 era), swimmers in Greece trained from September to mid-March, stopped swimming training for two months, and trained again from mid-May until the national championships in mid-July. During the 2020–2021 season, training started in September and stopped at the end of October; swimmers started training again in March and competed in mid-July (Figure 1).

### 3.2. Coaches’ Evaluation of the Training Process

**Figure 2:**
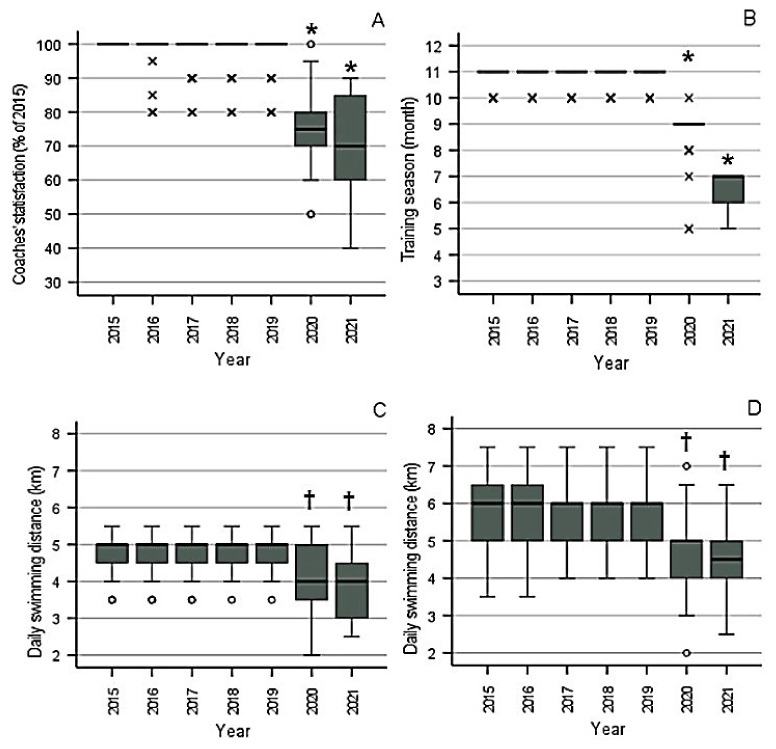
Coaches’ evaluation of the training process between 2015 and 2021. (**A**) Coaches’ satisfaction; (**B**) duration of the training season; (**C**) daily swimming distance during training of the short-distance swimmers; (**D**) daily swimming distance during training of the middle-distance swimmers. Each gray box represents the interquartile range, and the solid horizontal line represents the median. Whiskers are extended to the most extreme data point, which is no more than 1.5 times the interquartile range from the edge of the box (Tukey style). Circles represent outliers that are no more than three times the interquartile range from the edge of the box, and x marks represent outliers that are farther away. * Significantly different from all other years, † significantly different from 2015 through 2019 (all *p* < 0.001).

Analysis of coaches’ satisfaction with the training process through the Friedman test exhibited a significant effect of the year (*p* < 0.001, Figure 2A). Post-hoc analysis through the Wilcoxon test with Bonferroni correction showed that values in 2020 and 2021 were significantly different from those in all other years (*p* < 0.001). This included the comparison between 2020 and 2021, with scores of 75 (70–83)% and 70 (55–85)% (median and interquartile range), respectively.

The duration of the training season, as reported by the coaches, showed a significant effect of the year (*p* < 0.001, Figure 2B). As can be expected by the inspection of Figure 1, the training season was significantly shorter in 2020 and even more in 2021 compared to 2015–2019 (*p* < 0.001 for all pairwise comparisons). The median months of training dropped from 11 in 2015–2019 to 9 in 2020 and 7 in 2021.

There was also a significant effect of the year on the daily swimming distance during the training of the short-distance swimmers (*p* < 0.001, Figure 2C). The post-hoc analysis revealed that the daily swimming distances were shorter in the COVID-19 era, with a median of 4.0 km in 2020 and 2021, compared to all previous years, with a median of 5.0 km (*p* < 0.001).

Finally, the analysis of the daily swimming distance during the training of middle-distance swimmers revealed a significant main effect of the year as well (*p* < 0.001, Figure 2D). The swimming distance was shorter in 2020 and 2021 (with medians of 5.0 and 4.5 km, respectively) than in previous years, with a median of 6.0 km (*p* < 0.001).

### 3.3. Swimmers’ Participation in Championships and Performance

As can be seen in Table 1, the numbers of swimmers who achieved the qualifying times for the national championships in their category during the COVID-19 era were similar to those in previous years. Specifically, the average yearly number of swimmers for all categories in the 100-m freestyle event was 41 between 2015 and 2019 vs. 46 in 2020 and 48 in 2021. The corresponding numbers for the 400-m freestyle event were 28, 25, and 25.

**Figure 3:**
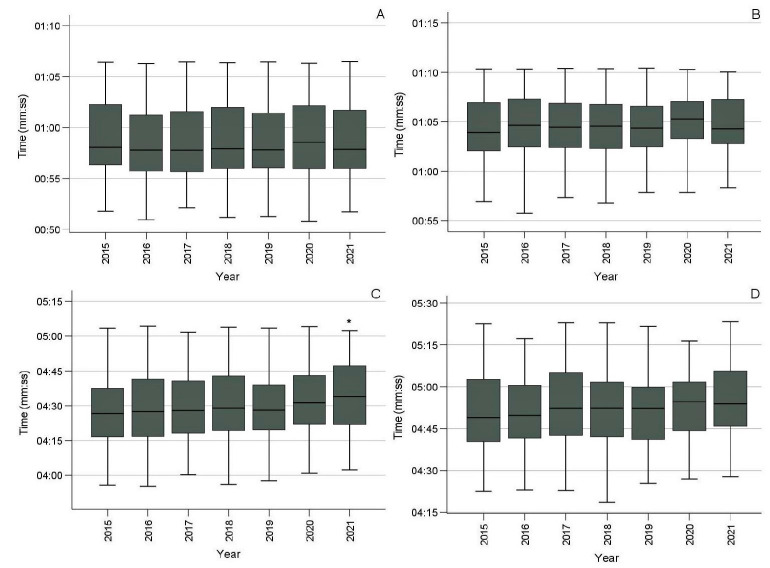
Performance of swimmers in seven championships: (**A**) males, 100 m; (**B**) females, 100 m; (**C**) males, 400 m; (**D**) females, 400 m, all freestyle. * Significantly different from 2015 (*p* = 0.001).

To facilitate and increase the power of the comparison of swimmers’ performance between championships, we decided to unify the records of all males in the 100-m event (rather than presenting each age category separately) and to do the same with females in the 100-m event, males in the 400-m event, and females in the 400-m event. The results are presented in Figure 3. The Kruskal–Wallis H test revealed a significant effect of year only in the 400-m freestyle event in males (*p* = 0.017). Post-hoc analysis through the Mann–Whitney U test with Bonferroni correction located the difference between 2015 and 2021 (*p* = 0.001), with the performance being inferior in 2021 by 7.24 s, or 2.7%.

To provide an additional measure of the changes in performance from 2015 to 2021, Figure 4 presents the winners’ records in each event and age category. It can be seen that records fluctuated over the years, without any consistent trend.

## 4. Discussion

The present study was triggered by the expressed fear of coaches that the performance of their swimmers in the Greek swimming championships during the COVID-19 era would be worse than in previous years because of the low annual training volumes. This is a justifiable concern, as research has shown that a break in swimming training of as little as five weeks decreased aerobic fitness [8]. Our analysis confirmed that coaches evaluated the training process with lower scores in 2020 and 2021, compared to 2015 through 2019. Nevertheless, most of the data regarding swimmers’ performance did not show any significant changes across years.

Despite the decrease in swimming training volume during the COVID-19 era, the number of swimmers who participated in the Greek swimming championships and met the HSF time standards for the 100-m and 400-m freestyle events did not change considerably (Table 1). We could not find any similar information in the scientific or non-scientific literature, that is, information that could reflect the impact of pandemic-imposed lockdowns on participation in athletic events, except for the stable number of participants in big international meetings. However, this was because those athletes had not been forced to stop their training.

**Figure 4:**
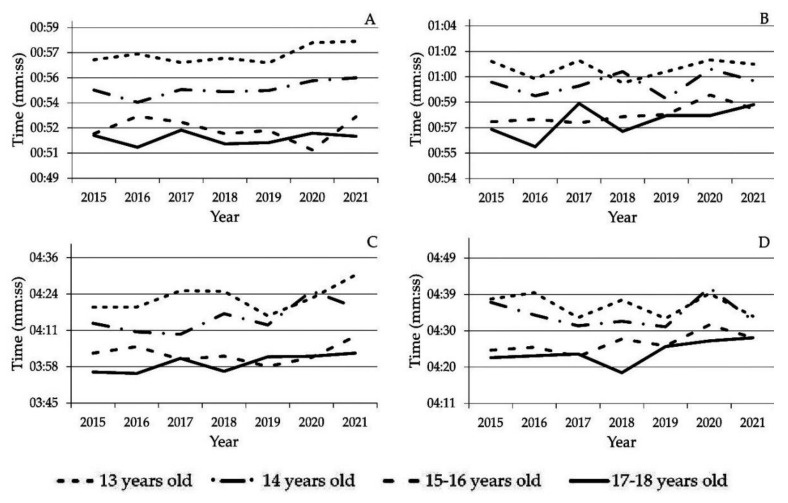
Winners’ records by age category in the freestyle events of 100 m in males (**A**), 100 m in females (**B**), 400 m in males (**C**), and 400 m in females (**D**) in the 2015 through 2021 championships.

The daily swimming training distances reported in the present study (with medians ranging from 4 to 6 km; Figure 2) are similar to those in other research studies [9,10,11,12], which have reported 3.6 to 6.4 km as common daily training distances for swimmers of the ages in question. However, other authors have suggested considerably higher volumes (around 5 to 8 km). Specifically, Sweetenham and Atkinson [13], and Vorontsov [14] maintained that it is necessary to achieve yearly training volumes of 1600 to 2200 km for sprinters and 1900 to 2600 km for middle-distance swimmers aged 13 to 18. Lower volumes were reported by Mujika and coworkers [5] for elite short-distance swimmers (1126 km), while, based on the median daily swimming distance of short-distance swimmers between 2014 and 2019 in the present study, a yearly training volume of 1292 km could be calculated. Equally lower than the recommendations by Sweetenham and Atkinson [13], and Vorontsov [14] for middle-distance swimmers was the median yearly training volume found in this study for 2014 to 2019 (1556 km). Our values for the COVID-19 era were even lower (776 km in 2019–2020 and 550 km in 2020–2021 for the short-distance swimmers, and 904 and 627 km, respectively, for the long-distance swimmers). Yet, the performance did not show any clear trend of improvement or deterioration during the period studied (Figure 3 and Figure 4), except for that in the 400-m event in males, which dropped in 2021.

It should be noted that the reduced yearly training volumes during the COVID-19 era were not due to reduced training frequency, but to abstention from swimming training for two periods of two and four months. Such interruptions in training during the competitive season were too long, and only the impact of common detraining periods, such as 4- to 10-week postseason breaks, which reduced the performance of young swimmers, have been studied [6,7]. No information, to our knowledge, exists in the literature about the impact of very long cessations of training during two consequence competitive seasons on swimming performance. Nevertheless, the ensuing return to training (which happened to have the same duration as the interruption) restored swimming ability, with the exception noted above. Therefore, it appears that two to four months of mixed dry-land and swimming training after a break of similar duration in swimming training was sufficient to restore performance in championships for these categories of swimmers in most cases. Other researchers have reached similar conclusions [15,16,17,18]. In addition, Costill, as cited by Chatard and Stewart [18], found that halving swimming training volume (from 8.7 to 4.5 km per day) over two competitive seasons resulted in improved swimming power and performance.

A limitation of this study is the presence of several confounding factors (different maturation, evolution, training experience, level, etc.) or even some specific characteristics of the swimmers in each age group, which were unknown and, thus, could not be entered in the statistical analysis. Another limitation is that, due to its retrospective nature, we could not have additional details of the training process, such as intensity, efficiency, and technique, which are considered components of quality rather than quantity [4]. Efficiency of training refers to the suggestion that swimmers can reduce training distance with no loss of endurance capacity if they perform faster swimming bouts [4]. Technique, on the other hand, emphasizes that performance is highly determined and predicted by technical factors [10,19]. Thus, it was possible that efficiency of training and technique differed between years. An additional limitation is the lack of data on dry-land training, which might also have differed from year to year.

## 5. Conclusions

The performance of young Greek swimmers was largely maintained during the COVID-19 era, despite the cessation of swimming training for two months in the 2019–2020 season and four months in the 2020–2021 season, which was followed by retraining of a similar duration. Therefore, coaches may not have to worry about breaks from swimming training, as long as swimmers maintain some dry-land training and return to the swimming pools for a sufficient time before competition.

## Figures and Tables

**Table 1:** Number of swimmers who were included in the analysis per category, event, geographical region of Greece, and year of championship.

Category		13 y Male		14 y Male		15–16 y Male		17–18 y Male		13 y Female		14 y Female		15–16 y Female		17–18 y Female	
Event		100	400	100	400	100	400	100	400	100	400	100	400	100	400	100	400
2015	S	24	31	33	34	16	15	21	15	29	14	20	14	22	20	15	10
	N	17	10	22	10	9	8	12	4	15	12	9	8	15	8	6	5
2016	S	19	25	35	16	20	13	24	20	27	16	33	13	25	21	13	7
	N	12	20	17	19	11	13	15	8	18	14	15	12	29	10	5	5
2017	S	20	31	35	26	13	17	27	9	30	20	25	19	35	17	13	9
	N	14	14	14	15	12	11	18	8	15	12	22	15	22	10	13	4
2018	S	24	25	28	27	15	20	26	15	25	21	37	15	24	24	11	10
	N	12	17	20	2	13	12	10	4	18	12	17	11	19	16	10	5
2019	S	29	30	32	28	18	14	23	12	2	21	39	22	37	29	12	3
	N	14	19	24	27	14	13	11	6	21	10	23	17	24	22	9	4
2020	S	26	37	32	24	12	18	25	7	40	19	35	14	36	23	9	6
	N	15	22	19	14	11	6	9	5	20	14	20	6	19	13	3	6
2021	S	30	33	47	16	22	20	22	10	21	16	49	18	39	21	12	8
	N	11	15	23	14	8	9	11	7	17	9	21	10	23	8	9	4

N, swimmers from Northern Greece; S, swimmers from Southern Greece; 100, 100-m freestyle; 400, 400-m freestyle.

## Data Availability

All data are available to interested parties upon request.
